# Movement and vocal intonation together evoke social referencing in companion dogs when confronted with a suspicious stranger

**DOI:** 10.1007/s10071-020-01401-3

**Published:** 2020-06-04

**Authors:** A. Salamon, J. Száraz, Á. Miklósi, M. Gácsi

**Affiliations:** 1 Department of Ethology, Eötvös Loránd University, Budapest, Hungary; 2 MTA-ELTE Comparative Ethology Research Group, Budapest, Hungary

**Keywords:** Dog–human interaction, Social referencing, Safe haven effect

## Abstract

Dogs have been claimed to engage in social referencing by responding in a way that corresponded with their owners' reaction in some contexts. We aimed to assess how owners’ actions affect family dogs’ behaviour in two ambiguous lifelike situations. In Experiment 1, two groups were tested; in the suspicious owner (SO) group, owners behaved suspiciously (*N* = 25), in the reassuring owner (RO) group, owners behaved in a reassuring manner (*N* = 28) facing a ‘threatening stranger’. The sitting owners provided voice intonation and body posture changes as cues for the dogs when the stranger entered the room. Dogs looked longer at the owners and stayed longer near them in the SO group but their tendency to approach the stranger did not differ between the groups. Although the owners’ behaviours seemed to have relevant effects on dogs’ responses, we note that these looking and proximity seeking behaviours might also be explained by reactions to the owners’ behaviour alone. In Experiment 2, all dogs (*N* = 19) were tested in both the SO and RO conditions in a slightly different procedure. Depending on the condition, owners took one step forward/backward and spoke happily/worryingly. The procedural differences and the larger distance between the stranger and the owner allowed the dog more time to perceive the behaviour of both the stranger and the owner, which made the distinction between alternative explanations for the dogs’ behaviour easier to interpret. Dogs spent more time behind their owners in the SO condition and more dogs approached the stranger in the RO condition. Dogs’ avoidance of the stranger when the owner behaved suspiciously and their tendency to approach the stranger only when the owner displayed positive emotions, can be best explained by social referencing.

## Introduction

Adjusting behavioural responses to that of important social partners in novel or ambiguous situations can be advantageous for young/inexperienced individuals. The process by which individuals rely on emotional displays of social partners in evaluating and responding to a novel, ambiguous stimulus or situation is called social referencing (Walden 1993). Obtaining information via observation and behaving accordingly may be considered as a first step of social learning (Heyes 1994) in such contexts. Social referencing may include different behavioural components, such as referential looking, which is defined as looking at an informant immediately preceded and/or followed by looking at a novel stimulus (Russell et al. 1997), and specific behavioural regulation, which is described as the subject’s behaviour consistently influenced by the (emotional) cues provided by the social partner upon encountering a new stimulus (Mumme et al. 1996; Russell et al. 1997). In many situations, however, simpler forms of social learning, such as stimulus enhancement, or behavioural synchronisation—exhibiting similar behaviour at the same time (Duranton and Gaunet 2016)—may also evoke similarly efficient behavioural regulation on the part of the naive subject. For example, young chimpanzees (*Pan troglodytes*) touched and interacted more with a remote controlled toy after observing their mother interacting with it, compared to when they faced the novel object alone (Tomonaga et al. 2004).

Social referencing has been extensively investigated in infant-parent dyads, the parent being a source of information to which the infant is paying attention. Most paradigms involved infants being presented with an ambiguous object, person or situation in the presence of the caregiver who expressed either happy or fearful facial expressions. In studies using novel or ambiguous objects, when the mother displayed a fearful facial expression, infants avoided an object (Sorce et al. 1985) or played less with ambiguous toys (Gunnar and Stone 1984). Infants were friendlier to a stranger when their mothers spoke positively to them about the stranger (Feinman and Lewis 1983), whereas infants, who observed anxious interactions between their mothers and the stranger, behaved anxiously towards the stranger (de Rosnay et al. 2006).

Merola and colleagues reported that dogs (*Canis familiaris*) alternated their gaze between their owner and a strange object (fan), but did not find behaviour regulation corresponding to that of their owners’ (Merola et al. 2012a, 2013). However, in another study dogs also adjusted their behaviours in a way that corresponded to their owners’ signals (Merola et al. 2012b). Further, the same study reported that social referencing may also occur when unknown people provide cues for the dogs. These observations were interpreted as dogs showing social referencing in ambiguous situations. Investigating the development of social referencing Fugazza et al. (2018) reported that 8-week-old dog puppies were more likely to approach a novel stimulus and interact with it in the presence of a human showing positive vocal and facial cues, compared to a human showing neutral vocal and facial cues. An hour later, when being alone, puppies regulated their behaviour according to the humans’ previous behaviour indicating that they had learnt about the stimulus in the presence of humans’ signalling.

However, there could be several different social mechanisms contributing to the behavioural change observed in a naïve individual.

1. In an ambiguous situation, naïve dogs may stay close to their owners even in the absence of any relevant emotional signal, simply because they apply a sort of freezing strategy (Walker et al. 1997) and/or due to the safe haven effect, that is, the owner provides security for the dog (Gácsi et al. 2013a; Cimarelli et al. 2016). These explanations have been supported by results showing that the increase in family dogs’ heart rate was lower when they had to face a threatening stranger in the presence of their owners compared to the condition when they were alone (Gácsi et al. 2013a).
2. In the case of emotionally loaded human vocalisations, dogs might react to their owner's emotional signal by approaching the signaller irrespective of the novel/ambiguous stimulus in the environment (e.g., dogs tend to look at or approach a crying person: Custance and Mayer 2012). Yong and Ruffman (2015) demonstrated that in such situations dogs may respond to the emotional content of the person’s communication without later connecting it to the novel/ambiguous stimulus. Thus social referencing does not necessarily result in social learning. Although dogs behaved depending on whether an unfamiliar experimenter displayed fear or happiness during an encounter with a small robot, when the dogs were left alone, there was no difference in dogs’ proximity to the robot depending on the emotion the human displayed previously.
3. Behavioural synchronisation could also play a role in the behaviour of the naïve individual. When movement cues are provided, i.e., stepping forward or backward, dogs may show behaviour synchronisation following the movements of their owner (Duranton et al. 2017a), which might be erroneously interpreted as a reaction to the novel stimulus. Recently Duranton et al. (2016) tested dogs’ reactions towards a stranger in the presence of their owners. In the positive condition, the owners took three steps forward (approach condition), whereas in the negative condition they took three steps back (retreat condition). The owners were not permitted to show any facial expressions, emotions or speak, while the stranger looked at the owners. Most dogs alternated gaze between the owner and the stranger, and they approached the stranger later in the retreat condition than in the approach condition. However, due to the experimental setup, dogs might alternate gaze between the stranger and the owner simply because the owner showed intense behaviours. This could have elicited looking behaviour even in the lack of the stranger and dogs’ moving together with their owners could likely be due to behavioural synchronization rather than social referencing.

The evolutionary function of all these social processes is to guide the young, inexperienced individual in novel/ambiguous situations, and make fast reactions and efficient social learning possible. In lifelike situations, for example, when the owner shows a complex response (vocalisation, movement, facial expression) to a frightening stimulus, these processes may work in combination.

Here, we aimed to extend the social referencing paradigm for dog-owner dyads modelling real life situations, when the owner provides complex responses. Our intention was to examine the overall reactions of dogs in such situations and not to assess the emergence of individual components of social referencing in more controlled and therefore unnatural scenarios. Since well-socialised companion dogs meet strangers in their everyday life in multiple places, we applied a stimulus that could be ambiguous enough to evoke social referencing. The ‘threatening approach’ paradigm (Vas et al. 2005) has been widely used to investigate family dogs’ responses to an ambiguous social stimulus. The name of the procedure is misleading; it refers to its differences from the ‘friendly approach’. Actually, the stranger does not display any direct threat, such as raising her hands or shouting, but silently and slowly approaches the dog while staring at it. Based on several studies, dogs show variable responses towards the stranger, ranging from displaying friendly behaviours (e.g., play bow: Győri et al. 2010), through neutral, and submissive behaviours to fear or aggression related behaviours (Vas et al. 2005; Gácsi et al. 2013b; Kis et al. 2014; Klausz et al. 2014). Thus the novel experimental conditions applied in our experiments were the stranger’s ‘threatening approach’ type of behaviour and the provision of lifelike complex behavioural responses on the part of the owner. So far, dogs’ reactions to strangers based on their owners’ cues have only been examined in situations when the owners provided only movement cues to the dogs (Duranton et al. 2016, 2017b).

To assess family dogs’ responses to their owners’ suspicious and/or reassuring reactions we carried out two experiments, which allowed for applying both between- and within-subjects designs and also to model two slightly different lifelike situations. In Experiment 1, the dogs faced the stranger with their owners in a context modelling an office situation where the owners were sitting at a fixed location during the encounter. Applying a between-subjects design, the owners displayed reassuring behaviours in the reassuring owner (RO) group and suspicious behaviours in the suspicious owner (SO) group with their actions limited (by the situation) to voice intonations and body posture changes when talking to the threatening stranger.

In Experiment 2, to avoid the effects of potential uncontrolled factors, dogs (different from those involved in Experiment 1) were tested in a within-subjects design so that the same dogs participated in both the RO and SO conditions. Dogs faced the stranger with their owners in a large hall modelling a street or public place (e.g., shopping malls), where the larger distances (compared to Experiment 1) allowed the dog more time to perceive and respond to the behaviour of both the stranger and the owner. Depending on the condition, owners made one small step towards or away from the threatening stranger and talked to her with a happy or worrying intonation, respectively.

We predicted that in such social situations, dogs would respond to the ambiguous stimulus according to their owners’ behaviour. Specifically, when the owner displayed worrying behaviours, dogs would show more avoidance towards the stranger and/or more proximity seeking with the owner, and in contrast, when receiving reassuring cues, dogs would approach the stranger.

## Experiment 1

### Methods

A written statement (PE/EA/3742-4/2016) was obtained from the Food Chain Safety and Animal Health Directorate Government Office based on the decision of the Scientific Ethic Council of Animal Experiments. According to this statement and the corresponding definition by law, this non-invasive observational study was not considered as an animal experiment, therefore it was allowed without the need for permission from the University Institutional Animal Care and Use Committee (UIACUC, Eötvös Loránd University, Hungary).

### Subjects

Fifty-five family dogs (mean age ± SE = 3.6 ± 0.5 years; 31 females and 24 males) and their owners (mean age ± SE = 31.0 ± 1.3 years; 48 women and 7 men) participated voluntarily, who were recruited through the database of the Family Dog Project, Budapest. Based on the owners reports, all dogs were well-socialised pets and we only included dogs in the experiment if they behaved in a friendly manner with an unfamiliar experimenter (other than the one who played the role of the stranger) before the test.

A between-subjects design was used; dogs were divided randomly into two groups. In the RO group 28 dogs were tested and analysed (mean age ± SE = 3.5 ± 0.5 years; 17 females and 11 males; mean age of owners ± SE = 30.4 ± 1.3 years; 24 women and 4 men owners; Table 1). From the SO group, two dog-owner dyads were excluded due to procedural mistakes during the test, so 25 dogs’ data could be analysed (mean age ± SE = 3.8 ± 0.6 years; 12 females and 13 males; mean age of owners ± SE = 31.9 ± 2.5 years; 22 women and 3 men owners; Table 1).

**Table 1:** The breed, sex and age (years) of the dogs tested in Experiment 1

Reassuring owner group			
ID	Breed	Sex	Age
Reassuring owner group			
1	Dalmatian	Female	3
2	Mongrel	Female	3
3	Mongrel	Female	3
4	Mongrel	Male	6
5	Mudi	Female	1
6	American pit bull terrier	Male	2
7	Belgian shepherd	Female	4
8	Kelpie	Male	9
9	Border collie	Female	8
10	Mongrel	Female	1
11	Mongrel	Male	10
12	German pointer	Male	4
13	Miniature poodle	Female	1
14	Mongrel	Male	1
15	Dachshund	Male	1
16	Cavalier King Charles spaniel	Female	3
17	Mudi	Female	1
18	Labrador retriever	Female	7
19	Golden retriever	Male	1
20	Greyhound	Male	1
21	Mongrel	Female	2
22	Border collie	Male	7
23	Mongrel	Female	2
24	Mongrel	Female	1
25	Mongrel	Male	2
26	Beagle	Female	6
27	Hungarian vizsla	Female	3
28	Hungarian vizsla	Female	5
Suspicious owner group			
29	Mudi	Female	7
30	Nova Scotia duck tolling retriever	Female	5
31	Border collie	Male	2
32	Mudi	Female	6
33	Mongrel	Male	7
34	Mongrel	Female	3
35	Bernese mountain dog	Female	4
36	English bulldog	Male	6
37	Shiba inu	Male	2
38	Beagle	Male	1
39	Mongrel	Female	1
40	Hungarian vizsla	Female	9
41	Mongrel	Male	3
42	Mongrel	Female	4
43	Pekingese	Male	5
44	Dachshund	Female	1
45	Beagle	Male	6
46	Border collie	Male	1
47	Mongrel	Male	2
48	Mongrel	Male	2
49	Fox terrier	Female	1
50	Border collie	Male	11
51	Border collie	Female	2
52	Golden retriever	Female	2
53	Mongrel	Male	1

### Procedure

**Fig. 1:**
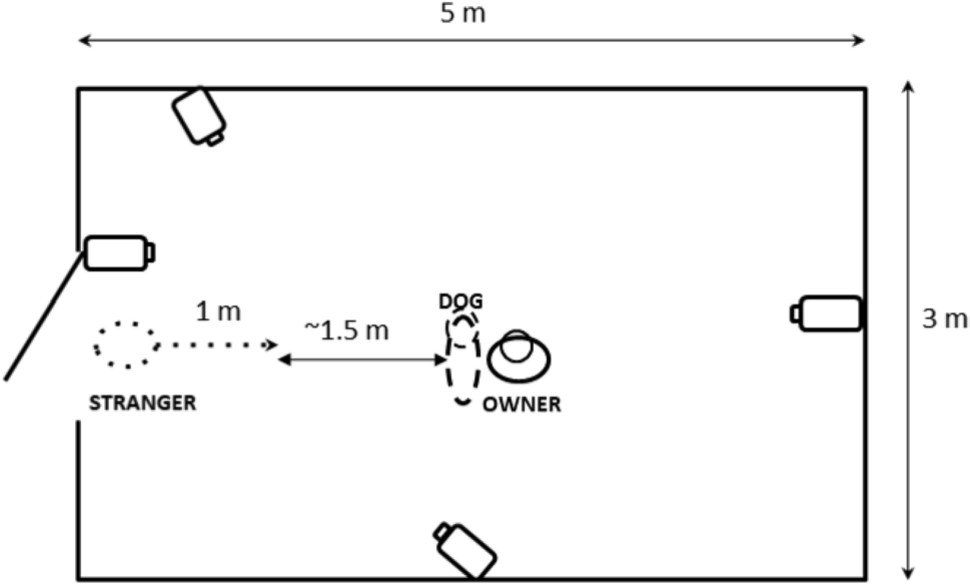
The setup of Experiment 1. The dotted arrow represents the movements of the stranger during the test. The point of the dotted arrow represents the final position of the stranger. The smaller circles on the owner and dog represent their heads in the beginning of the test. The stranger enters the door on their left side

**Fig. 2:**
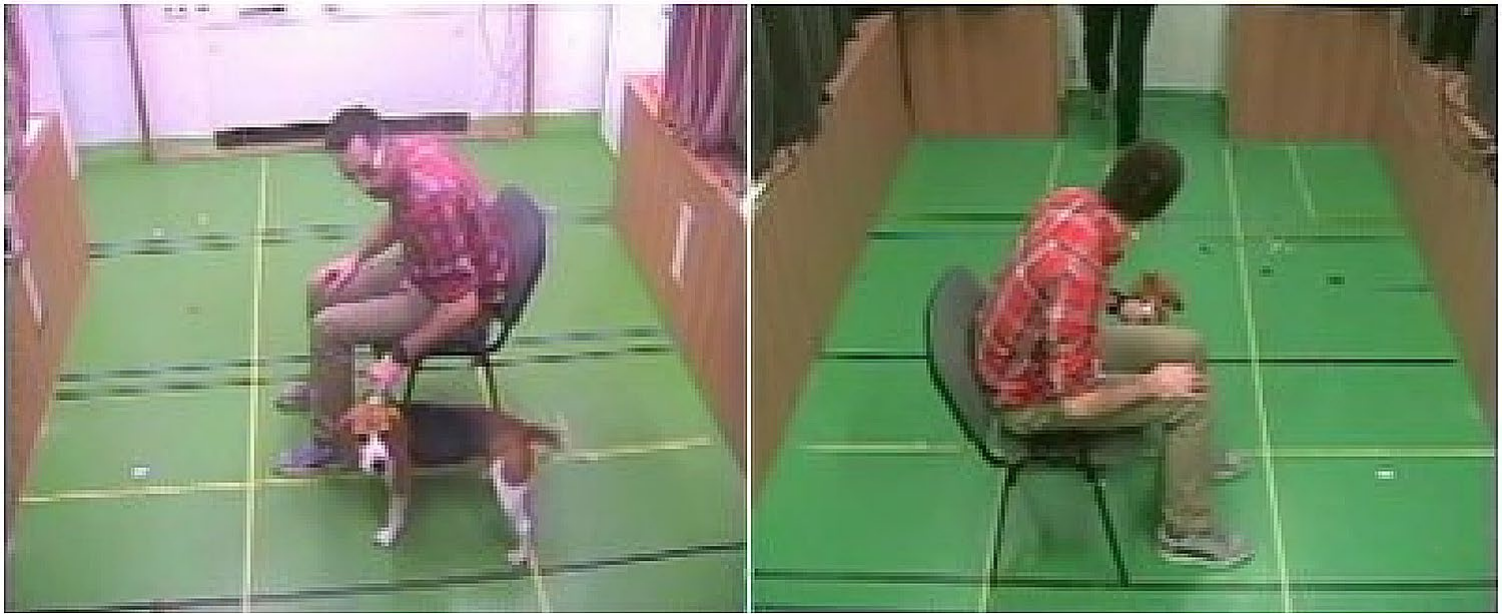
The starting position of the owner, dog and stranger in Experiment 1 from the two camera positions

The subjects were tested individually in an unfamiliar room at Eötvös Loránd University (Fig. 1). Before the test began, the dog was free to explore the room for 2 min. The owner was seated in the middle of the room, about 2.5 m from the door, sideways to the door, because we did not want to influence the dog’s position by the orientation of the owner’s body. At the beginning of the test, the seated owner held the dog by its collar (Fig. 2). The unfamiliar female experimenter (same for all dogs) knocked on the door to catch the dog’s attention and entered the room. She began to move as soon as she could make eye contact with the dog. In that moment, the owner released the collar and began the required actions (voice intonation and posture change) depending on the condition. From this moment the dog’s behaviour was observed for 25 s.

In the RO group, the owner was instructed to say pleasant words in a relatively high pitched voice (e.g., “Hello, it is nice to see you. It has been a long time…”) and lean towards the stranger. We asked the owners to display behaviours as spontaneously as possible (within the constraints of the protocol) towards the stranger (as if they were in a real life situation). In the SO group, the owner was instructed to gasp, talk suspiciously (e.g., “Dear me! What do you want! Don’t come closer…!”), and lean away from the stranger. In both conditions, the owners were told not to use the dog’s name and potential commands such as “look, go, come, touch”. In both groups the owner was asked to look at the stranger (not at the dog) during the entire test, thus he/she did not alternate his/her gaze between the dog and the stranger as it happened in some other studies with novel objects (e.g., Merola et al. 2012b; Fugazza et al. 2018).

The stranger took small, slow steps towards the dog with slightly bent upper body, while keeping a steady gaze without any vocal communication. She moved in a straight line (see Fig. 1), but looked at the dog whichever way the dog was moving. The movements of the stranger covered 1 m during the test. For safety reasons the stranger was asked to stop and break eye-contact, should the dog start to growl or bark (*N* = 1) until the end of the test.

After the test was finished, in both groups the dog was called by the stranger in a friendly voice and was stroked in order to provide relaxation from the test situation.

### Behavioural observations and data analysis

The test was recorded with four video cameras (see Fig. 1). The records were coded with Solomon Coder (beta 12.09.04, Copyright 2006–2008 by András Péter) using the following behavioural variables: duration and latency of looking at the owner (s), time spent near the owner (within the distance of the dog’s body length) (s), whether the dog approached the stranger within the distance of the dog’s body length (yes/no) and the latency of approach (s). The latency variables included only data from animals that performed the given action (looked at the owner, approached the stranger), to compare the timing of these actions in the two conditions (see e.g., Fugazza and Miklósi 2015), as maximum latency data could have been redundant with the occurrence variables. Inter-observer agreement was assessed on 15 entire tests between two coders and high Cohen’s kappa values were found (> 0.86 for all behaviours).

The normality of the distribution was checked using Shapiro–Wilk tests. Since the data was not normally distributed, Mann–Whitney tests were used to compare the duration and latency of looking at the owner, time spent near the owner, and the latency of approach. Wilcoxon Signed Ranks test was used to compare the latency of looking at the owner and the latency of approaching the stranger within each group (in case of dogs that looked at the owner and approached the stranger). Chi-square test was used to compare the number of dogs that approached the stranger in the two groups. Statistical analyses were run on SPSS 24 (IBM Corp., New York).

## Results

**Fig. 3:**
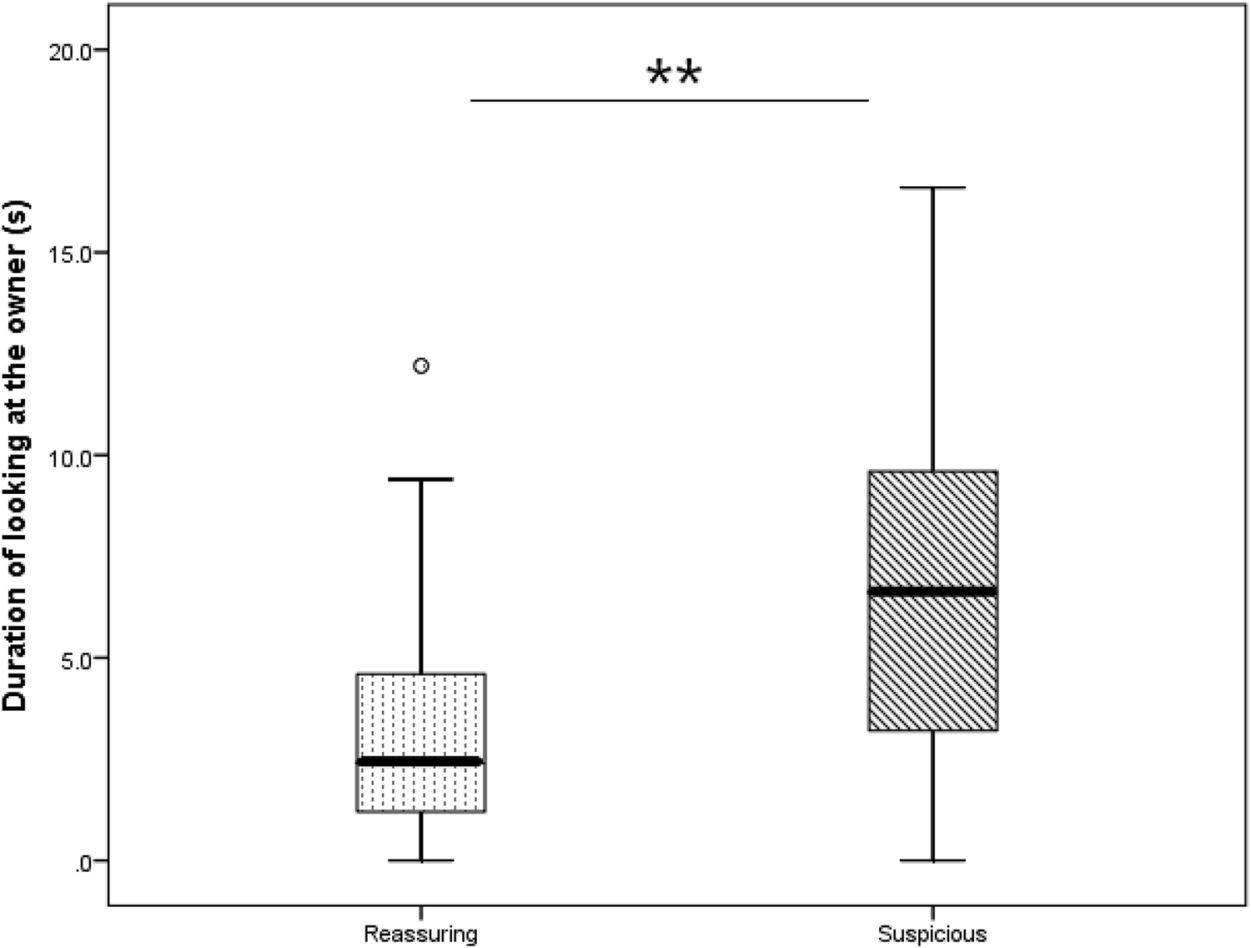
The time the dogs spent looking at the owner in the two groups (median, quartiles, whiskers show highest and lowest values no greater than 1.5 times the interquartile range). The open dot represents an outlier. (**p < 0.01)

Though in both groups most dogs looked at least once at their owners (in the RO group 26 dogs out of 28 and in the SO group 24 dogs out of 25), dogs in the SO group looked significantly longer at their owners compared to the RO group (RO group median: 2.4 s, SO group median: 6.6 s; *U* = 192.5, *p* = 0.005; Fig. 3). The latency of looking at the owner did not differ between the groups (RO group median: 3.2 s, SO group median: 3.3 s; *U* = 297.5, *p* = 0.778).

**Fig. 4:**
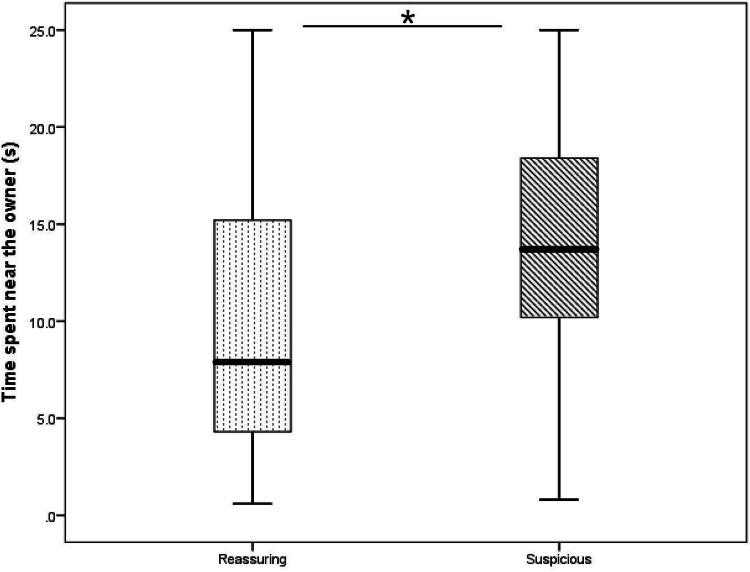
The time the dogs spent near the owner in the reassuring or suspicious groups (median, quartiles, whiskers show highest and lowest values no greater than 1.5 times the interquartile range). (*p < 0.05)

Dogs spent more time near the owner in the SO group than in the RO group (RO group median: 7.8 s, SO group median: 13.6 s; *U* = 230.5, *p* = 0.033; Fig. 4).

In both groups most dogs approached the stranger (RO group: 82%, SO group: 88%) and there was no difference in the latency of approach between the two groups (the median was 2 s in the RO group and 1.7 s in the SO group; *U* = 184.5, *p* = 0.118). In the RO group dogs approached the stranger before they looked at the owner (*z* = − 2.643, *p* = 0.008), but there was no significant difference between the latency of approaching the stranger and the latency of looking at the owner in the SO group (*z* = − 0.922, *p* = 0.357).

## Discussion

Most dogs looked at the owner during the test, which suggests that they were seeking information from the owners regarding the approaching stranger similarly to the study of Duranton et al. (2016). As at the beginning of the test all dogs were looking at the stranger (see procedure), their looking at the owner within a few seconds may be considered as referential looking. Alternatively, dogs may have looked at their owners simply because the owner was speaking and/or their talk was emotionally loaded or just the audition attracted the dog’s attention. We also note, that despite referential looking is considered a major element of social referencing, it is also claimed that vocal intonation alone can serve as a social referencing cue (Colbert-White et al. 2018). In our test situation, breaking eye contact with a threatening stranger may have negative consequences, and the unequivocal emotionally loaded vocal intonation of the owner could be sufficient social referencing cue to rely on.

**Fig. 5:**
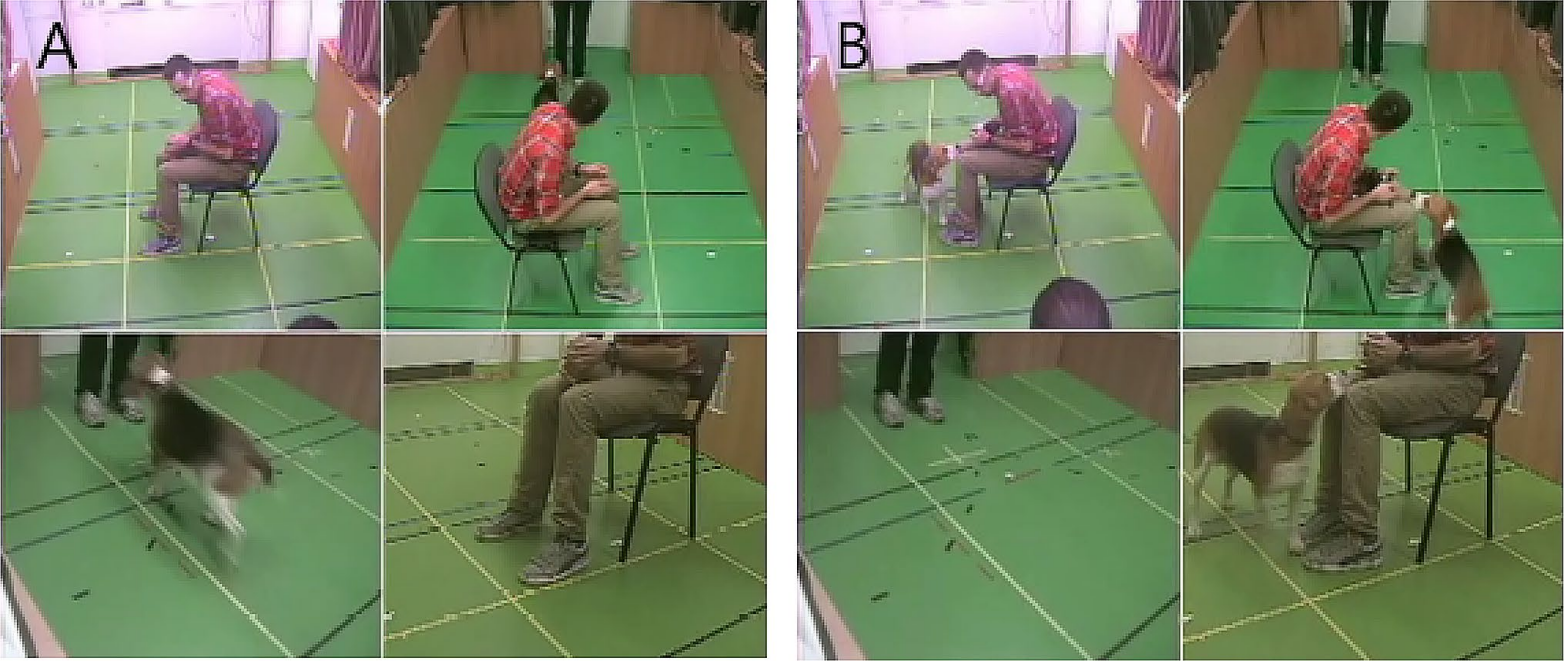
Illustration of owner’s posture reaction and a typical example of dog behaviour in the suspicious owner condition. **a** 1 s after the start dog approaches the stranger. **b** 7.6 s after the start dog is near the owner. The four images show the same moment from different angles

Dogs looked at their owners longer and stayed longer near the owners when he/she acted suspiciously, which could indicate that dogs were more affected by the negative emotional communication (Fig. 5b). Indeed, besides being a source of information, the owner can also serve as a source of security for the dog. However, it is also possible that the owner’s worrying vocalisation evoked more attention and approaching by the dog, just because the owner’s communication was more stressful for the dogs (Yong and Ruffman 2015; Huber et al. 2017).

In this procedure, most dogs approached the stranger in both the RO and SO groups, which does not support our assumptions related to the owners’ effect as social informant, and also raise questions about the efficiency of the ‘threatening stranger’ paradigm in this context. Though previous studies reported that companion dogs typically showed more avoidance behaviour and less approach to a stranger displaying a threatening compared to a friendly (Vas et al. 2005) or playful (Győri et al. 2010) approach, in these studies dogs were tethered to a tree and were not able to run to the stranger who approached from about 5 m away.

A plausible explanation for our results could be given by analysing the observed behaviour sequences: considering the rather low approach latency (≤ 2 s in both conditions), most of these well-socialised family dogs could have responded by approaching the stranger before they could recognise either the threatening behaviour of the stranger or the owner’s specific emotional cues (see Fig. 5a). This is supported by the looking latency results, as dogs tended to look at their owners after they had approached the threatening stranger, which suggests that they might only have realised the strange behaviour of the unfamiliar person during the encounter.

In Experiment 1, the between-subjects design allowed for assessing the responses of the dogs in one condition without the interference of the other condition. However, this way we could not control for potential influencing factors (and their interaction effects), such as age, gender, breed, keeping condition, training status, general attitude with strangers, attachment to owner, which might have an effect on the behaviour of dogs.

Therefore in Experiment 2, dogs (different from those involved in Experiment 1) were tested in a within-subjects design so that the same dogs participated in both the RO and SO conditions. This test was conducted in a large hall, modelled a street or public place (e.g., shopping malls), where the larger distances (compared to Experiment 1) allowed the dog more time to perceive and respond to the behaviour of both the stranger and the owner.

## Experiment 2

### Methods

#### Subjects

In this experiment 25 dog-owner dyads participated based on the owners’ will to volunteer. Based on the owners reports, all dogs were well-socialised pets and we only included dogs in the experiment if they behaved in a neutral/friendly manner with an unfamiliar experimenter (other than the one who played the role of the stranger) before the test. None of them had participated in Experiment 1. Six dogs were excluded from the analysis due to procedural errors during the test. The remaining 19 dogs were all 1 years of age or older (mean age ± SE = 3.6 years ± 0.6; 9 females and 10 males; Table 2). All dogs received basic obedience training, six dogs were therapy dogs and five were trained in agility.

**Table 2:** The breed, sex, age (years) and training (1: basic obedience, 2: therapy, 3: agility) of the dogs tested in Experiment 2

ID	Breed	Sex	Age	Training
1	Chinese crested dog	Female	4	1
2	Groenendael	Female	4	1, 2
3	Groenendael	Female	1	1, 2
4	Chinese crested dog	Male	2	1, 2
5	German shepherd	Male	6	1
6	Cairn terrier	Male	4	1, 3
7	German shepherd	Female	1	1
8	Miniature dachshund	Male	3	1
9	Mongrel	Female	1	1
10	Border collie	Male	4	1, 3
11	Border collie	Male	9	1, 2, 3
12	Golden retriever	Male	5	1, 2
13	Golden retriever	Female	3	1, 2
14	Shetland sheepdog	Male	2	1
15	Shetland sheepdog	Male	1	1
16	Groenendael	Female	3	1
17	Mongrel	Female	2	1
18	Shetland sheepdog	Male	11	1, 3
19	Toy poodle	Female	2	1, 3

All owners were female (mean age ± SE = 32.2 ± 2.6 years).

#### Procedure

In Experiment 2, a within-subjects design was used; all dogs participated in both conditions in a counterbalanced order. Similarly to Experiment 1, owners were asked to talk to the stranger in a different tone of voice depending on the condition. We predicted that when the owner displayed worrying behaviours, dogs would show more avoidance towards the stranger and/or more proximity seeking with the owner, while when receiving reassuring cues, dogs would rather approach the stranger.

**Fig. 6:**
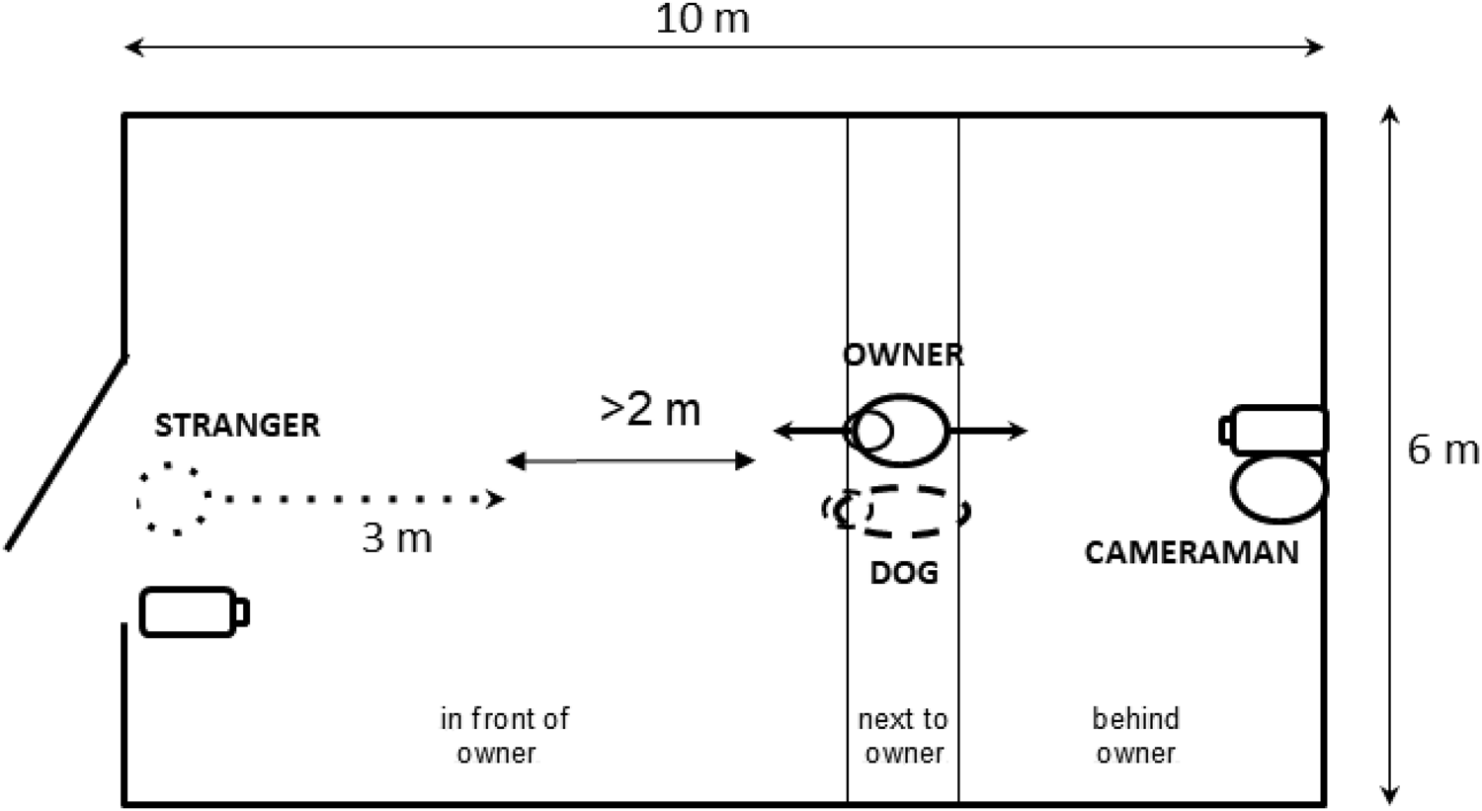
The setup of Experiment 2. The dotted and black arrows represent the movements of the stranger and the owner respectively during the test. The point of the dotted arrow represents the final position of the stranger, while the black arrows represent the final position of the owner depending on the condition (the distance between these two points was at least 2 m). The small circles represent the heads of the owner and the dog

At the beginning of the test the owners stood in the room (see Fig. 6) and when the stranger established eye contact with the dog, they moved one step forward in the RO condition and one step back in the SO condition. In addition to the emotionally loaded vocalisation (and potential facial expressions that we could not control for), we considered the one small step approach or retreat as behaviours conveying the emotional state of the owner.

The dogs were tested in a large unfamiliar room (see Fig. 6). During the test four participants were present in the room: the dog, the owner, a cameraman, and an unfamiliar female experimenter (different from stranger in Experiment 1) playing the role of the stranger. We wanted to make the stranger look more suspicious, as in Experiment 1, over 80% of dogs approached the stranger in both conditions. Therefore, similarly to the study of Kerepesi et al. (2015) the stranger had a hood on, which—based on experts’ view and personal experience (M.G.)—can make a person look suspicious for dogs. Before the test began, the dog was free to explore the room for 2 min, while the cameraman explained the procedure to the owner (the stranger was not in the room yet).

At the start of the testing session, the owner stood with the dog beside her, on a loose leash. The stranger knocked on the door to catch the dog’s attention and stepped in the room. She started to move after establishing eye contact with the dog. When the stranger moved ahead, the owner dropped the leash and began the actions (stepping and speaking with specific voice intonation). The owners were asked to look at the stranger (not at the dog) during the entire test, thus they did not alternate their gaze between the dog and the stranger during the test as during other studies (e.g., Merola et al. 2012b; Fugazza et al. 2018).

The stranger took small, very slow steps towards the dog with slightly bent upper body, while keeping a steady gaze without any vocal communication (see Vas et al. 2005). The stranger moved in a straight line (see Fig. 6), but oriented her head towards the dog at all times to maintain eye-contact. The movements of the stranger covered 3 m, which was about halfway to the owner. This distance was maintained to allow for differentiating between the dogs’ moving together with the owner versus approaching the stranger. For safety reasons the stranger was asked to stop and break eye-contact, if the dog should start to growl or bark (*N* = 3). The test lasted 15 s. After the test was finished, the dog was called by the stranger in a friendly voice and was stroked in order to provide relaxation from the test situation.

After the first condition, the owner took the dog outside for a 5-min break. Then the second condition followed with the same stranger and same procedure except the owner’s reactions. Using the same stranger seemed better in terms of not presenting an additional variable into the procedure and was validated by the results of Vas et al. (2008), which showed that dogs adjust their behaviour depending on the approach of the stranger (friendly vs. threatening) even in repeated trials.

#### Behavioural observations and data analysis

The test was recorded with two video cameras, one placed on a tripod near the door where the stranger entered, the other held by a cameraman who followed the movements of the dog. To analyse the dog’s behaviour, the videos were coded using Solomon Coder. Compared to Experiment 1, in Experiment 2 we measured slightly different behavioural variables due to the different set up and procedure: latency of looking at the owner(s); duration of time spent in front of, next to, and behind the owner(s) (see Fig. 6), whether the dog approached the stranger (within the distance of the dog’s body length) (yes/no), and the latency of approaching the stranger (including data only from dogs that approached the stranger) (s).

To assess the reliability of the coding, inter-observer agreement was calculated on ten entire tests with two different people coding them. High Cohen’s kappa values were found for all behaviours (> 0.81 for all) showing high consistency in the coding.

To test the effect of the conditions and order of presentation, Generalized Linear Models were used with normal distribution and log link function, where the fixed factors were the condition and the order; and their interaction was also included. In case of the approach variable binomial distribution and logit function were used. All the statistics were run on SPSS 24 (IBM Corp., New York).

## Results

All dogs looked at their owners at least once in both conditions, however neither condition (Wald = 0.139, *p* = 0.709) nor order of the conditions (Wald = 0.052, *p* = 0.819) or their interactions (Wald = 1.601, *p* = 0.206) had an effect on the latency to look at the owner (the median was 1.2 s in both the RO and SO conditions).

Condition had a significant effect on the position of the dog; dogs spent more time behind their owners in the SO condition compared to the RO condition (Wald = 5.225, *p* = 0.022). The difference in the duration of time spent in front of their owner was not significant (Wald = 3.198, *p* = 0.074) and there was no effect of condition on the duration of time spent next to the owner (Wald = 0.156, *p* = 0.693). Dogs’ position was affected by neither the order of the conditions (in front of owner Wald = 0.839, *p* = 0.36; next to owner Wald = 1.717, *p* = 0.19; behind owner Wald = 0.001, *p* = 0.97), nor the interaction between the order and the condition (in front of owner Wald = 0.033, *p* = 0.856; next to owner Wald = 0.014, *p* = 0.904; behind owner Wald = 0.021, *p* = 0.886).

**Fig. 7:**
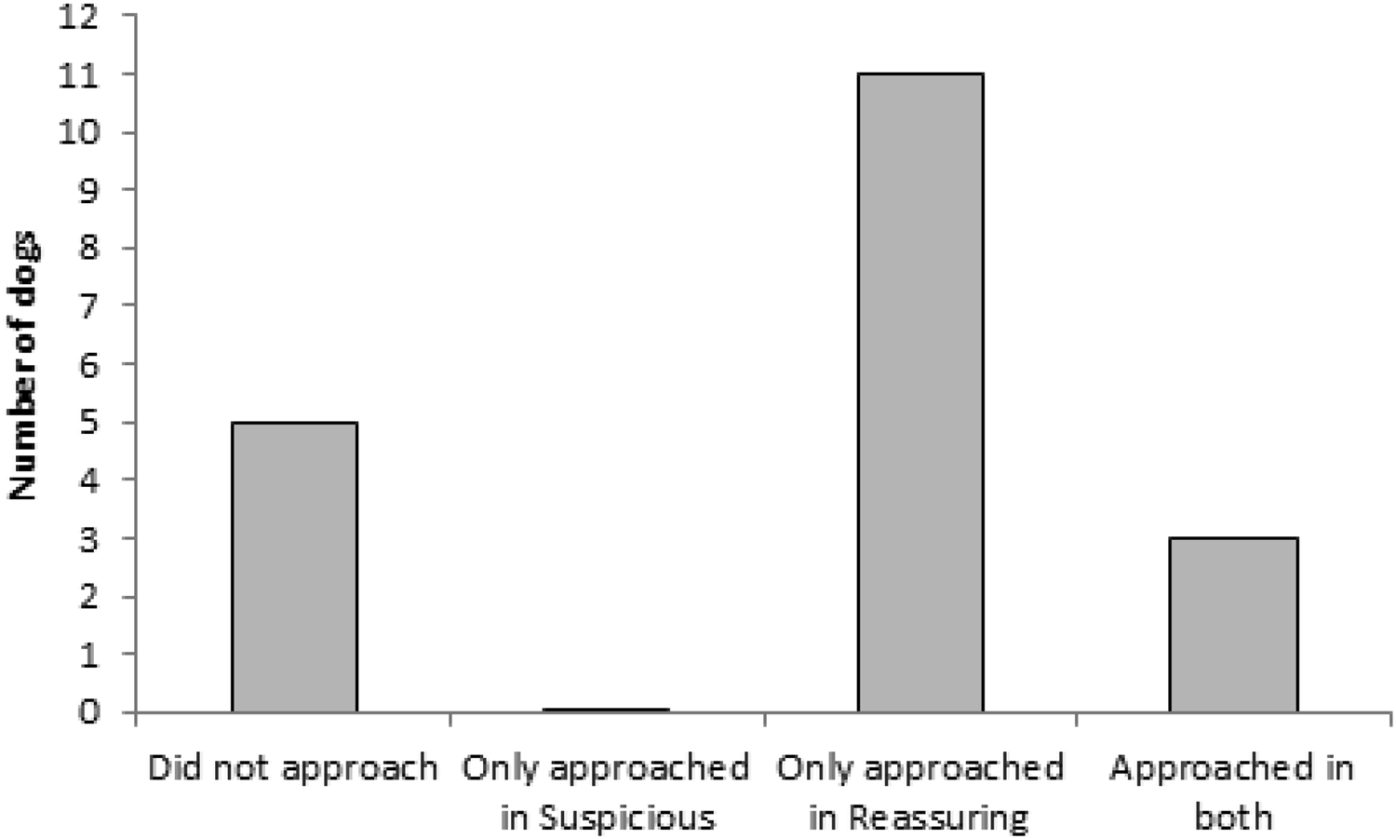
The number of dogs (out of 19) that did not approach and those that approached the stranger in either one or both conditions

Condition had a significant effect on the dog’s approach to the stranger (Wald = 10.565, *p* = 0.001); in the RO condition 14 dogs approached the stranger, while in the SO condition only 3 dogs did so. Neither the order of the condition (Wald = 0.01, *p* = 0.92) nor the interaction between the order and the condition had an effect on the approach of the stranger (Wald = 2.032, *p* = 0.154). Further, five dogs did not approach the stranger in either conditions, whereas three dogs approached the stranger in both conditions (two of them were therapy dogs), thus 11 out of 19 dogs showed behaviours corresponding to the owner’s behaviour (Fig. 7).

The latency of approach was only meaningful in those three dogs that went to the stranger in both conditions. The data show a tendency of the latency being lower in the RO condition compared to the SO condition (3.4 s vs. 4.5 s; 4.4 s vs. 8 s; 3.8 s vs. 11.2 s).

## Discussion

In Experiment 2, in the SO condition dogs did not only seek the owners’ proximity, which could also be explained by the safe haven effect, but retreated behind the owner. The longer duration of time dogs spent behind the owner indicates a tendency to avoid the stranger rather than just seeking comfort from the owner.

In this setup, the distance between the endpoints of the stranger and the owner were more than 2 m, so the dog had to leave the owner to approach the stranger. In comparison, in the Duranton et al. (2016) study, dogs could remain close to their owners when the owner took three regular steps towards the stranger in their approach condition. In that case, the distance between the endpoints of the stranger and the owner was only 1 m. This way dogs could approach the stranger while still remain near the owner (within body length), therefore the owner could serve as a safe haven for them.

In this experiment, a few dogs approached the stranger in both conditions, which could be explained either by not being sensitive to the threatening type of the approach or, in the case of therapy dogs, by their experience with unusually behaving strangers. Some dogs did not approach the stranger in either condition, which could be due to their high sensitivity to the threatening signals of the stranger or their lack of interest in the stranger (as we only included dogs that behaved in a friendly manner with unfamiliar people). In the above cases the owners’ reactions did not seem to have a significant effect (or their actions were not believable for the dogs), though based on the latency data of dogs that approached the stranger in both conditions, even though they could have been affected by the owners behaviour. Most importantly, all dogs that showed condition specific responses, approached the stranger only in the RO condition. This indicates that the owners’ reassuring behaviour encouraged the dogs to approach the stranger and/or their suspicious behaviour inhibited the dogs from doing so.

## General discussion

Our aim was to study how owners’ reactions to a threatening stranger affect the behaviour of their dogs. The two experiments modelled lifelike situations, which allowed for social referencing between dog and owner, however we also considered alternative explanations for the correspondence in their behaviours.

In Experiment 1, though the owners’ behaviour significantly affected the dogs’ responses, dogs’ preference to stay near the owner longer when their owners behaved suspiciously, can also be interpreted by other processes than social referencing. The observed difference in the dogs’ position in the two conditions could be due to increased attraction to the owner because of her worrying vocal intonations (Yong and Ruffman 2015). Our results could also be explained by the owners’ safe haven effect (Cimarelli et al. 2016) and not by the avoidance of the stranger. It is possible that from the owner’s suspicious vocalization dogs recognized the threat, but did not link it to the entrance of the stranger, but instead retreated to the safe haven of the owner.

The short distance between the dog and the stranger at the start of the experiment could explain why most dogs approached the threatening stranger so quickly (within 2 s) in both groups. In a previous similar study carried out in a room (3 × 5 m), most dogs showed stress responses to the stranger, but the dog and owner were positioned at the far end of the room (Gácsi et al. 2013a). We also note that so far in all tests using the ‘threatening approach’ paradigm dogs were tethered, so that dogs had more time to watch the behaviour of the stranger, while in our off leash contexts they could approach the stranger before they could realise the threatening aspects of her behaviour. This methodological difference could also contribute to the smaller effects in our test.

In summary, dogs preferred to stay close to their sitting owners when the owners behaved suspiciously, but in this situation the owners’ vocal intonation and posture changes (in the absence of stepping movement) did not evoke similar social referencing in dogs to what was demonstrated in infant studies (Feinman and Lewis 1983; de Rosnay et al. 2006).

In Experiment 2, we expected more profound differences in the dogs’ responses between the two conditions, due to the owners’ more distinct behavioural reaction (taking a step towards or away from the stranger instead of leaning towards or away from her) and the larger distance between the stranger and the dog-owner team, which allowed more time for the dog to process the social situation. Indeed, the results gave more support to the utilization of social referencing in this setup.

Dogs stayed longer behind their owners when the owners’ reaction was suspicious towards the stranger, which suggests that the dogs’ responses were influenced not just by the behaviour of the owner but can also be linked to the stranger’ approach. Moreover, dogs were more likely to approach the stranger in the RO condition, which could be attributed to the encouraging behaviour of the owner and cannot be explained simply by behaviour synchronisation, because the owner only made a small step towards the stranger but did not approach her.

So far studies investigating the occurrence of social referencing in dogs have provided diverse results, which could be due to both methodological and theoretical factors. Though it is crucial to investigate complex social processes in different contexts, it should also be noted that even in the case of simpler socio-cognitive abilities, the smallest differences in the applied methodology can significantly influence the findings and lead to erroneous interpretations (see e.g., Pongrácz et al. 2013). The fact that even applying a threatening and not just novel stimulus did not elicit a relatively uniform basic reaction from dogs (over 80% of dogs approached the stranger in both conditions in Experiment 1; 16% of dogs approached the stranger in both and 26% in neither conditions in Experiment 2) suggests that we should always address the many confounding factors in such studies. In general, the social behaviour of adult dogs and infants proved to be similar in many respects, e.g., the development of social attraction and individualized attachment, flexible behavioural and emotional synchronization, social learning, and rule following abilities, pronounced sensitivity to the human’s communicative intent and to the referential character of human cuing (Topál et al. 2009), but we can expect larger variability in adult dogs’ spontaneous responses towards novel/ambiguous stimulus compared to that of infants.

In studies with humans, it has been suggested that the process of social referencing during infancy can be particularly important in the development of social anxiety (Murray et al. 2008). Similarly, if applied consistently when rearing a puppy, such processes may shape the dog’s behaviour without explicit training (Fugazza et al. 2018). Interestingly, the resemblance between dogs’ and owners’ personality (e.g., Kis et al. 2012) was interpreted partly by the probability that the owner’s everyday spontaneous reactions were shaping the dog’s typical behaviour (Turcsán et al. 2012), for which process social referencing might be an efficient mechanism. Thus, the owners’ spontaneous reactions in ambiguous situations may have lasting effects on the puppy and investigating such a mechanism further could help understand, for example, how some dogs develop fearful or aggressive behaviours towards strangers or other dogs. Such potential impacts and practical implications need to be further investigated.
